# Advancing Drug Safety in Drug Development: Bridging Computational Predictions for Enhanced Toxicity Prediction

**DOI:** 10.1021/acs.chemrestox.3c00352

**Published:** 2024-05-17

**Authors:** Ana M. B. Amorim, Luiz F. Piochi, Ana T. Gaspar, António J. Preto, Nícia Rosário-Ferreira, Irina S. Moreira

**Affiliations:** † Department of Life Sciences, University of Coimbra, Calçada Martim de Freitas, 3000-456 Coimbra, Portugal; ‡ CNC-UC—Center for Neuroscience and Cell Biology, University of Coimbra, Calçada Martim de Freitas, 3000-456 Coimbra, Portugal; § CIBB—Centre for Innovative Biomedicine and Biotechnology, University of Coimbra, Calçada Martim de Freitas, 3000-456 Coimbra, Portugal; ∥ PhD Programme in Biosciences, Department of Life Sciences, University of Coimbra, Calçada Martim de Freitas, 3000-456 Coimbra, Portugal; ⊥ PURR.AI, Rua Pedro Nunes, IPN Incubadora, Ed C, 3030-199 Coimbra, Portugal; # PhD Programme in Experimental Biology and Biomedicine, Institute for Interdisciplinary Research (IIIUC), University of Coimbra, Casa Costa Alemão, 3030-789 Coimbra, Portugal

## Abstract

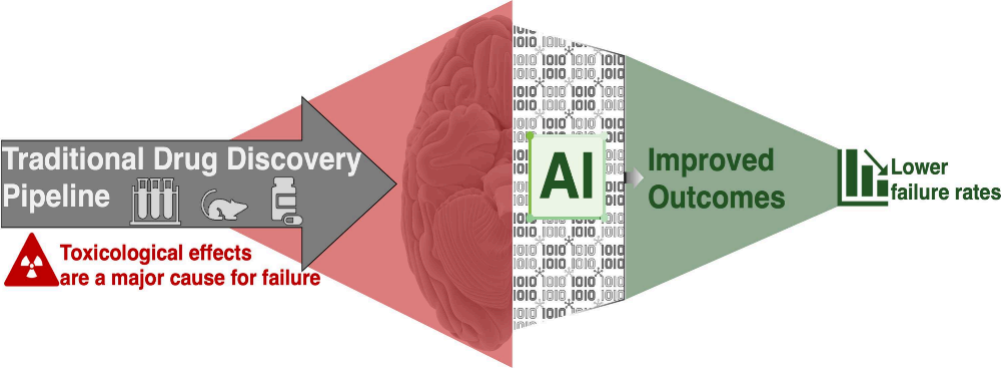

The attrition rate of drugs in clinical trials is generally quite high, with estimates suggesting that approximately 90% of drugs fail to make it through the process. The identification of unexpected toxicity issues during preclinical stages is a significant factor contributing to this high rate of failure. These issues can have a major impact on the success of a drug and must be carefully considered throughout the development process. These late-stage rejections or withdrawals of drug candidates significantly increase the costs associated with drug development, particularly when toxicity is detected during clinical trials or after market release. Understanding drug-biological target interactions is essential for evaluating compound toxicity and safety, as well as predicting therapeutic effects and potential off-target effects that could lead to toxicity. This will enable scientists to predict and assess the safety profiles of drug candidates more accurately. Evaluation of toxicity and safety is a critical aspect of drug development, and biomolecules, particularly proteins, play vital roles in complex biological networks and often serve as targets for various chemicals. Therefore, a better understanding of these interactions is crucial for the advancement of drug development. The development of computational methods for evaluating protein–ligand interactions and predicting toxicity is emerging as a promising approach that adheres to the 3Rs principles (replace, reduce, and refine) and has garnered significant attention in recent years. In this review, we present a thorough examination of the latest breakthroughs in drug toxicity prediction, highlighting the significance of drug-target binding affinity in anticipating and mitigating possible adverse effects. In doing so, we aim to contribute to the development of more effective and secure drugs.

## 1 Introduction

In drug development, ensuring safety and efficacy is a paramount goal that requires comprehensive understanding of the intricate interplay between drugs and biological systems. At the heart of this interaction lies the critical role of proteins involved in virtually every biological process, including metabolic pathways, DNA replication and modification, signaling cascades, and immune responses. These processes are crucial not only for maintaining cellular function but also for understanding pathogenic mechanisms, disease progression, and the development of diagnostic tools.^1^ Proteomics, a term introduced by Wilkins,^2^ is a field of study that covers areas such as protein–protein interactions (PPIs), identification of protein expression profiling, and mining of the proteome and is key in the identification and validation of drug targets. Proteomic data and technologies are required for the identification and validation of drug targets, biomarker discovery, determination of drug efficacy and toxicity, and exploration of their mechanisms of action.^3−5^ However, the identification of potential therapeutic targets for the successful development of safe and effective drugs is fraught with challenges, particularly in predicting and mitigating toxicity. The dual aspects of pharmacodynamics (PD),^6^ the study of a drug’s effects on the body, encompassing its mechanisms of action, and pharmacokinetics (PK),^7^ which examines how the body affects a drug through processes such as absorption, distribution, metabolism, and excretion (ADME), together represent two critical aspects of drug research that are of great interest. Therefore, safety assessment requires parallel consideration of the intrinsic activity (PD) of a drug and its behavior within an organism (PK). This comprehensive approach is essential for predicting potential toxic effects and optimizing therapeutic efficacy.

Along with the many stages of drug development, an in-depth description of molecular compounds is essential for successful identification of promising innovative drugs. Compound characterization involves the study of the structural, physicochemical, biochemical, and pharmacokinetic properties of a drug, as well as its toxicity.^8^ The measure of harm caused by a particular substance to an organism, tissue, or cell is referred to as toxicity. The toxicity level of a drug can be influenced by several factors, including the duration and dose of exposure, route of administration, chemical shape and structure, and individual variations in human response.^9^ The prediction of drug toxicity is essential for drug development. Regulatory agencies such as the Food and Drug Administration (FDA) need to approve drugs so that they can enter the market. To achieve this, the drugs must have certain safety and efficacy standards. Furthermore, to fully characterize a substance, the safety of a molecule is inspected by recurrent examinations of drug–drug interactions, reactive metabolites, nonspecific targets, cardiotoxicity, mutagenicity, cytotoxicity, and teratogenicity.^8^ Toxicity has been estimated to be responsible for the withdrawal of approximately one-third of drug candidates, and is a major contributor to the high cost of drug development, particularly when it is not recognized until late in clinical trials or postmarketing.^10^ Toxicity and safety assessments are vital in drug discovery because forecasting toxicity enables the avoidance of undesirable effects of a drug, enhances its safety, and reduces the overall cost of drug development. However, existing experimental approaches, including time-consuming and costly procedures such as in vitro assays, animal studies, and clinical trials, are still limited in their effectiveness. The use of animal models raises problems in terms of extrapolation of animal data to humans, which is not always straightforward owing to species differences.^11^ Only 43–63% of toxicity predictions using rodent and nonrodent models match when extrapolated to humans and less than 30% when predicting adverse drug effects in target organs.^12^ Moreover, the use of animal models raises ethical concerns and emphasizes the need for alternative methods.^13,14^ Owing to the importance of this subject in the pharmaceutical industry, better computational methods should be developed to enhance the accuracy of predictive models and to construct accurate models that encompass the complexity of biological systems and accurately predict toxicity. Interdisciplinary collaboration, innovative technologies, data sharing, and the development of alternative testing methods are key to advancing the establishment of computational frameworks for determining drug toxicity.

The pursuit of novel therapeutics that balance efficacy and safety remains a central challenge in pharmaceutical innovation. Central to this endeavor is the sophisticated evaluation of drug efficacy and toxicity, which are two pivotal themes that dictate the trajectory of drug development. Anchoring this evaluation is the dynamic and intricate relationship between drug-target binding affinity (DTBA) and the range of possible toxicity outcomes, which is critical for assessing a drug’s clinical viability. DTBA serves as a vital indicator of how well a drug interacts with its target, essential for achieving intended therapeutic outcomes. However, the implications of this interaction go beyond efficacy to encompass safety concerns, highlighted by drug-target interactions (DTIs). The specificity and selectivity of DTIs can significantly affect the safety profile of a drug. Although high specificity is desirable for therapeutic promise, the possibility of off-target effects introduces risks that may diminish the benefits of the drug. This dual nature of DTIs, wherein the potential for both healing and harm, calls for a nuanced understanding and precise prediction of these interactions to navigate the fine balance between efficacy and safety. Moreover, on-target toxicity, in which drug-target interactions lead to unwanted side effects, and polypharmacology, which targets multiple pathways in complex diseases, add to the challenge of forecasting drug toxicity. Here, the cumulative effect of interactions with multiple targets raises the possibility of increased toxicity, underscoring the need for sophisticated predictive tools. Artificial intelligence (AI), a transformative force poised to redefine the paradigms of drug development, can be used to integrate vast data sets encompassing drug structures, target proteins, and toxicity profiles, enabling the prediction of adverse effects with unprecedented accuracy. By uncovering patterns and correlations beyond traditional methodologies, AI enhances predictive capabilities, offering a window into the complex interplay between drugs and biological systems. This predictive insight is not just about avoiding adverse effects, but also steering the drug design process toward safer, more effective therapeutic solutions.

**Figure 1:**
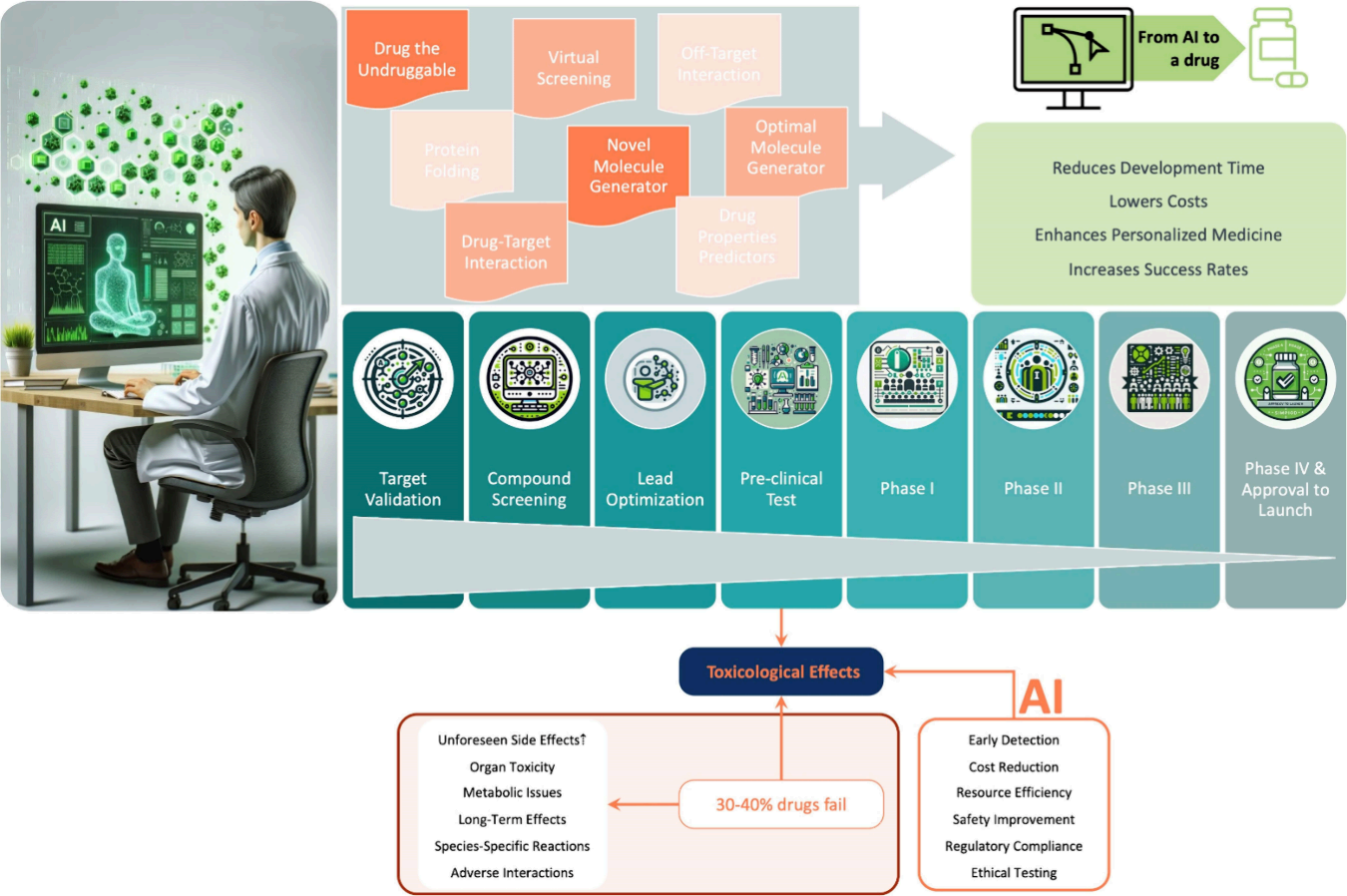
AI-driven drug development and toxicity forecasting, highlighting the use of AI in enhancing each stage of the drug development pipeline from target validation to approval and underscores its pivotal role in improving efficacy and safety by predicting toxicological risks.

This review focuses on cutting-edge methodologies that harness AI to predict drug toxicity, highlighting the critical role of DTBA in these evaluations. By marrying the intricate details of DTBA with AI’s predictive analytics, we aim to advance the safety assessment of drug candidates, weaving together efficacy and a forward-looking perspective on toxicity. Investigating DTBA’s dual function as both an indicator of efficacy and a predictor of potential toxicity allows us to examine how AI not only improves our understanding of drug interactions but also leads to the future generation of therapeutics. By examining this convergence of DTBA and AI, we can see that it is not just a technical accomplishment but also a critical step forward in achieving more effective and safe therapeutic interventions, ultimately leading to better patient care and public health outcomes (Figure 1).

## 2 How Can Artificial Intelligence Impact and Benefit the Drug Discovery Pipeline?

AI is a discipline that employs mathematical models and algorithms inspired by aspects of human cognitive functioning, such as learning and decision making,^15^ to develop technological tools to solve complex problems.^16^ AI has a wide range of applications in drug development, from screening to molecular design. To conduct drug screening using AI, models for predicting compound toxicity, bioactivity, and physicochemical properties have been constructed,^16^ contributing to the optimization of drug design and speeding up hit and lead recognition. Molecular design involves forecasting of drugs and target structures, de novo design by iteratively constructing drugs and testing their properties when interacting with the binding site, and the framework of drug target interactions (DTIs).^16,17^ Furthermore, AI is highly useful for drug repurposing to identify new therapeutic targets.^16^

Although AI approaches have proven pivotal in the prediction of drug toxicity, it is crucial to acknowledge the inherent limitations and experimental challenges associated with traditional methods. As previously mentioned, despite their longstanding contribution, conventional experimental assays for assessing drug toxicity often face constraints, such as high costs, time consumption, and ethical considerations.^10^ Recognizing these shortcomings, the implementation of AI approaches for drug toxicity prediction is key to efficient drug development. In recent years, the use of machine learning (ML) and deep learning (DL) has led to improvements in drug toxicity predictions through the development of numerous tools and methodologies.^10^ Accordingly, computational models for predicting drug toxicity have gained significant importance owing to their promising ability to reduce the money and time costs of large-scale experimental assays for assessing different toxicity end points. Methods with improved accuracy and efficiency provided by AI can help identify potentially toxic effects or harmful compounds prior to human clinical trials, ultimately resulting in time and cost savings.^18^ Indeed, the latest improvements are possible owing to advances in computational frameworks for retrieving relevant information from chemical structures and characteristics,^19^ and a range of methodologies have been used to forecast toxicity, including different types of neural networks, quantitative structure activity relationships (QSAR) tools, and molecular docking.^10,18^ For example, according to Luechtefeld’s findings,^20^ contemporary methodologies that integrate QSAR with artificial intelligence have proven highly effective in categorizing compounds across 19 different hazard categories, employing 74 labels as target features. These categories include acute toxicity (dermal, inhalation, oral), hazards to the aquatic environment (acute and chronic), sensitization (skin or respiratory), corrosion, irritation, and other significant concerns, such as carcinogenicity, reproductive toxicity, and environmental hazards. This comprehensive classification capability significantly enhances the safety evaluation process by providing information on potential hazards, thereby enhancing the development, approval, and use of safer compounds. These approaches successfully classified 87% of the evaluated compounds, surpassing the 81% success rate achieved by conventional in vivo tests. In light of their potential, in silico approaches should be considered the preferred choice for cutting-edge classification, prioritized over conventional techniques, and numerous general models for the simultaneous prediction of multiple toxicity end points and/or absorption, distribution, metabolism, excretion and toxicity (ADMET) features,^21−23^ as well as more specific models that focus on the prediction of specific toxicity effects, have been developed in recent years.^24−26^

**Figure 2:**
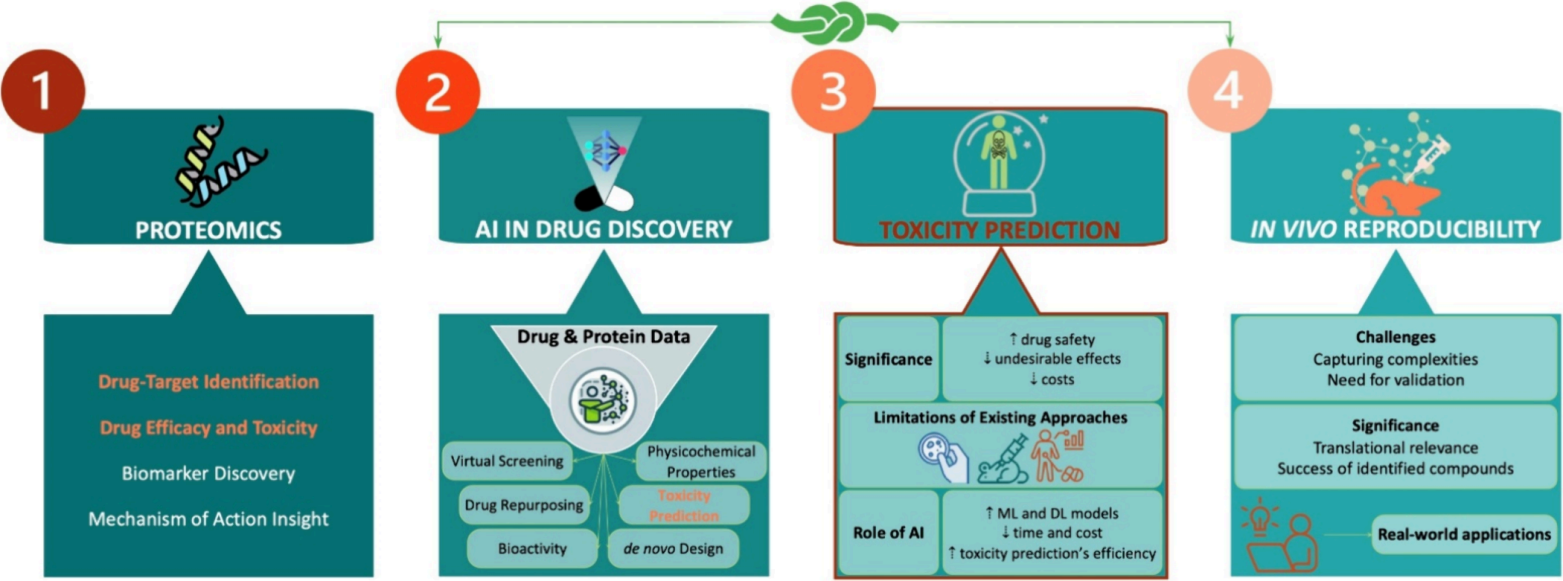
Confluence of drug development and predictive technologies, illustrating the key aspects of drug development, highlighting (1) the role of proteomic data, (2) applications of artificial intelligence (AI) in drug discovery, (3) the Significance, limitations, and role of AI in toxicity prediction, and (4) the significance and challenges of in vivo reproducibility.

Despite the significant impact of AI on drug discovery, the crucial role of in vivo studies remains, serving as a cornerstone in determining the translational relevance and effectiveness of the identified compounds. The in vivo environment presents a complex array of variables and challenges that are difficult to fully replicate using computational methods alone. Factors such as metabolism, pharmacokinetics, and unforeseen interactions within the host organism underscore the necessity for thorough in vivo validation. Integrating AI-driven insights with in vivo experiments offers a more holistic understanding of the therapeutic value of a compound, bridging the gap between computational predictions and practical applications in the nuanced realm of drug development. This synergy enhances the practical assessment of pharmacological impact, bioavailability, possible adverse effects, and overall therapeutic efficacy of a drug in a living system. Such in vivo evaluations are vital to understand the effects of drugs on different organs and tissues, thereby ensuring their safety and effectiveness. Furthermore, corroborating experimental findings with computational predictions strengthens the credibility and translational viability of the drug discovery process, marking a significant advancement in this field^16,27^ (Figure 2).

## 3 Advances in the Prediction of Toxicity End points

Toxicity end points are pivotal parameters for evaluating the safety of substances, particularly in the pharmaceutical and chemical industries. Tens of end points are relevant to drug toxicity identification. Here, we referred to five primary end points for toxicity prediction using algorithms in the field of AI: Lethal dose 50% (LD50), drug-induced liver injury (DILI), human ether-a-go-go-related gene (hERG) inhibition, carcinogenesis, and Ames mutagenesis, as there has been an increased focus on researching these end points more frequently.^10^ These end points provide crucial insights into different aspects of drug toxicity, enabling comprehensive evaluation and encompassing diverse biological responses, serving as measurable indicators of potential harm, and aiding risk assessment and regulatory compliance. LD50 provides insights into the potential lethality of a compound, DILI aides in the identification of hepatotoxicity risks, hERG inhibition helps in assessing the potential to cause adverse cardiac effects, while carcinogenicity offers perspectives into the long-term health risks associated with exposure to a compound and, Ames Mutagenicity furnishes understanding on compound’s ability to cause genetic mutations.^10,12,28^ Indeed, models benefit from utilizing well-established end points, which facilitate the incorporation of vast data sets with valuable information across different species, from rodents to humans. By scrutinizing these end points, it is possible to gauge their adverse effects on living organisms, enabling the identification and mitigation of potential hazards. The integration of these end points into toxicology studies forms a comprehensive framework for predicting and mitigating risks, thereby ensuring the development of safer products for human and environmental well-being. However, assays to identify these end points often require time, particularly at larger scales. As such, the correct development and use of several AI tools developed for the prediction of toxicity end points allows researchers to substantially decrease the time and workload required by narrowing the compounds of interest. Determining the validity of these predictions using different models, especially in vivo, is still key to ensuring proper scientific conduct.

LD50, for example, is a standard end point in toxicology, representing the amount of compound that leads to the death of half of the organisms after treatment in relation to an untreated control.^29^ It is usually one of the first end points of drug toxicity to be experimentally determined, along with the assessment of acute toxic reactions following administration via a given route of exposure. The LD50 of drugs is often reported as a measure of acute oral toxicity in rodents; thus, most predictive models are trained using oral toxicity data from mice and/or rats. However, some models do not specify which animal the data comes from or the route of exposure to the chemical, which limits the potential applications of these models. Although some models have considered interspecies variability,^30^ the majority of available algorithms rely exclusively on data from rodents and a single route of administration. Therefore, models that can utilize existing drug data to accurately predict acute toxicity and account for interspecies variability are highly valuable.

The significance of in silico tools has become more pronounced in the evaluation of tissue-related toxicity, particularly in cases of DILI and hERG-based cardiotoxicity. Given the role of the liver in the metabolism and excretion of several xenobiotics, it is frequently a target of injury caused by administered drugs and/or their metabolites. Drug-induced hepatotoxicity is one of the most common causes of acute liver failure in the U.S. and one of the most frequent causes of drug withdrawal.^31^ DILI can be classified as either acute or chronic, depending on the pathology, and can occur through various mechanisms. Thus, it is a key factor to be assessed early in drug development and highlights an opportunity for the employment of in silico tools. Toxicity, such as the heart level, is also a key parameter that is often determined during early toxicity assays. The hERG gene encodes the alpha subunit of the Kv11.1 potassium channel, which can be targeted and blocked by small molecules, which may lead to cardiac arrhythmia and fatal cardiotoxicity.^26^ Given that many drugs have been withdrawn because of their hERG-based cardiotoxicity, evaluation of hERG-blocking activity has become essential in drug development.^32^ There are different in vitro strategies for determining the cardiotoxic potential of hERG compounds; however, screening of many drugs is both time-consuming and expensive.^26^ As a result, in silico approaches have become appealing alternatives for determining hERG toxicity.

Carcinogenicity is a commonly discussed toxicity end point, owing to its significant impact on human health. Substances that cause cancer are classified as carcinogens and can cause cancer through various mechanisms, such as disruption of DNA damage repair or epigenetic changes.^33^ This enhanced carcinogenic potential has led to the withdrawal of several drugs from the market.^34^ As a result, algorithms capable of predicting the carcinogenic profiles of existing and new compounds can be valuable tools in drug development.

Similarly, the Ames test is an in vitro assay that assesses the potential of a compound to cause genetic mutations in *Salmonella typhimurium*. This is performed by exposing the bacteria to the compound and observing whether it results in the bacteria being able to produce the amino acid histidine and multiply.^35^ Therefore, the Ames test is often used to evaluate the mutagenic properties of compounds, and is sometimes related to their potential to cause cancer. It is important to note that these compounds can cause cancer through other mechanisms, and their mutagenic potential and carcinogenic effects should be assessed. Despite being relatively straightforward, it is recommended that the Ames test should be performed using at least five different strains of *S. typhimurium* and varying concentrations of the compound.^36^ Thus, as the workload scales, along with the number of evaluated drugs, models capable of accurately determining the Ames mutagenicity of compounds can provide valuable information during the drug development process.

### 3.1 Data Sources

Since multiple ML and DL algorithms have been employed to forecast drug toxicity and demonstrate proficiency in identifying harmful drugs and their probable adverse effects, it may be challenging to remain informed about the latest advancements, making a comprehensive examination of the present data sources, algorithms, and models beneficial. The development of these models requires high-quality data, coupled with curation processes. Data quality is a direct determinant of the performance and reliability of AI models. Furthermore, the accessibility of high-quality data enhances the capacity of AI systems to generalize their knowledge, thereby enabling them to make better predictions in real-world scenarios.^10^ Fortunately, several public toxicology databases contain data that can be used to collect relevant information. It is critical that the metadata and assays through which these metrics are experimentally obtained are clearly described to avoid any potential errors during the selection of data sets for the development of algorithms. However, many available databases suffer from inconsistencies in data generation arising from imprecise sources or integration from varied origins, leading to informational or formatting discrepancies. Moreover, the absence of standardized guidelines and uniformity in data generation and publication results further complicate data integration and analysis. Advancements in toxicological testing guidelines and protocols have enabled the prediction of potential toxic effects; however, transferring these data from controlled studies to real-world applications can be complex. A significant amount of toxicity research depends on animal studies, which may not always accurately reflect human responses due to differences in biology, and there are gaps in the data. A critical issue is the lack of extensive toxicological studies on many substances, highlighting the significant deficits in our understanding of their safety profiles. These shortcomings may result from inadequate testing, absence of standardization in data generation and dissemination, or rapid introduction of new chemicals that exceed the capacity of the databases to update. Additionally, the rate of chemical innovation and utilization may surpass the capacity of toxicological databases to provide current and relevant information. Considering these constraints, users of toxicological databases must critically evaluate the origin, completeness, and relevance of the data to their needs, while acknowledging the inherent constraints and biases inherent in the information provided.^37^

The Chemical Effects in Biological Systems (CEBS) database has provided public information on toxicogenomics data, including study design and timeline, clinical chemistry and histopathology findings, and microarray and proteomics data, since 2002.^38^ This database has been updated over the years; however, similar to other databases, the heterogeneity of available data stemming from contributions from various sources, such as academic research, industry, and regulatory submissions, may lead to inconsistencies within the data set. Another publicly available database is the Comparative Toxicogenomics Database (CTD), which stores manually curated information on chemical-gene/protein interactions and chemical-disease relationships. It was released in 2004 and is still regularly updated, currently boasting over 2.8 million curated chemical-gene interactions, along with over 3.3 million chemical-disease associations.^39^ Additionally, the Distributed Structure-Searchable Toxicity Database (DSSTox) is a valuable resource that connects data on chemicals, such as bioassays and physical properties, to their specific chemical structures, aiding in predicting toxicology.^40^ PubChem, which contains freely accessible chemical information, is a large and constantly updated database. It gathers information on over 115 million compounds and 308 million substances, along with bioassays, bioactivity, and gene and protein information.^41^ Toxicity Forecaster (ToxCast), a program developed by the United States Environmental Protection Agency (EPA), comprises data on approximately 1800 chemicals from a broad range of sources. Moreover, Toxicity Reference Database (ToxRefDB) compiles in vivo data from more than 5900 studies that either adhere to established guidelines or closely resemble guideline standards, covering a comprehensive set of over 1100 chemicals. This updated database contains information on study design, chemical administration, dosage, treatment effects, and adverse outcomes.^42^ Furthermore, to register a substance with the European Chemicals Agency (ECHA) established in 2007, a dossier that includes standard information and the inherent toxic properties of the substance is required, such as development toxicity, no-observed adverse effect levels (NOAELs), and lowest observed adverse effect levels (LOAELs).^43^ Finally, the Chemical Structure Database (ChemSpider) stores information on chemical structures, identifiers, properties (experimental and predicted), spectra, crystallography, and images. Currently, it provides access to 128 million chemical structures from over 270 data sources. However, the choice of a database often depends on the objectives of a given project. For example, CEBS, DSSTOX, PubChem, and ChemSpider contain general toxicity information related to health and chemical properties. For the development of toxicity prediction models, other databases such as CTD, ToxCast, and ToxRefDB can be more useful because they highlight information on different toxicity metrics. In developing models for toxicity prediction, leveraging multiple databases can further enhance comprehensive data coverage and address limitations in chemical structures, biological targets, toxicity end points, and experimental data inherent to single databases. However, data integration poses challenges related to measurement units, data formats, and experimental conditions. Therefore, focusing on data consistency and quality is central to this process.

#### 3.1.1 Evolution of Toxicity Prediction and Challenges

The ToxCast EPA in vitro to in vivo Challenge, overseen by TopCoder in 2014, aimed to create a model predicting the lowest effect level concentration based on in vitro measurements. The development of the Rank-I model underscores the importance of integrating biological knowledge, the role of pharmacokinetics, and the limitations of in vitro assays, particularly in scenarios where metabolic activation is significant. Additionally, it addresses another challenge regarding the transparency and reproducibility of modeling practices.^44^ Another program, the Tox21 Challenge,^45^ underscored the critical importance of accurately predicting nuclear receptor end points, which is a key aspect of toxicity assessment. In this context, DeepTox^46^ has emerged as a notable success, delivering an exceptional performance that outperforms other computational methods. By leveraging a deep neural network (DNN) architecture, DeepTox distinguished itself not only through its superior predictive capabilities but also by addressing the challenge of model interpretability. This is a crucial factor in enhancing our understanding of how these models arrive at their conclusions, thereby providing deeper insights into the mechanisms underlying the toxicity predictions. This dual achievement of high performance and improved interpretability positions DeepTox is a significant advancement in the field of computational toxicology.^46^ Nevertheless, predicting toxicity based on in vitro or in vivo assessments may lack consistency and accuracy in predicting clinical toxicity. A recent study addressed this limitation by introducing a framework that provides improved and explainable clinical toxicity predictions, with a low amount of animal data used. The authors demonstrated the advantages of DL, and more concretely, multitask models and transfer learning to predict toxicity across in vitro, in vivo, and clinical platforms. These findings suggest a reduced reliance on in vivo data for clinical toxicity prediction, accompanied by a posthoc contrastive explanation method to enhance interpretability.^47^ Overall, these advances mark a promising trajectory for reshaping the future of predictive toxicology.

### 3.2 AI-Based Models

A clear distinction between models for predicting drug toxicity is the generality or specificity of the predicted end points. As previously mentioned, numerous toxicity end points have been investigated, and several AI-based methods have been developed to predict various end points and ADMET features, including toxicity, whereas other models specialize in predicting a single end point.

**Figure 3:**
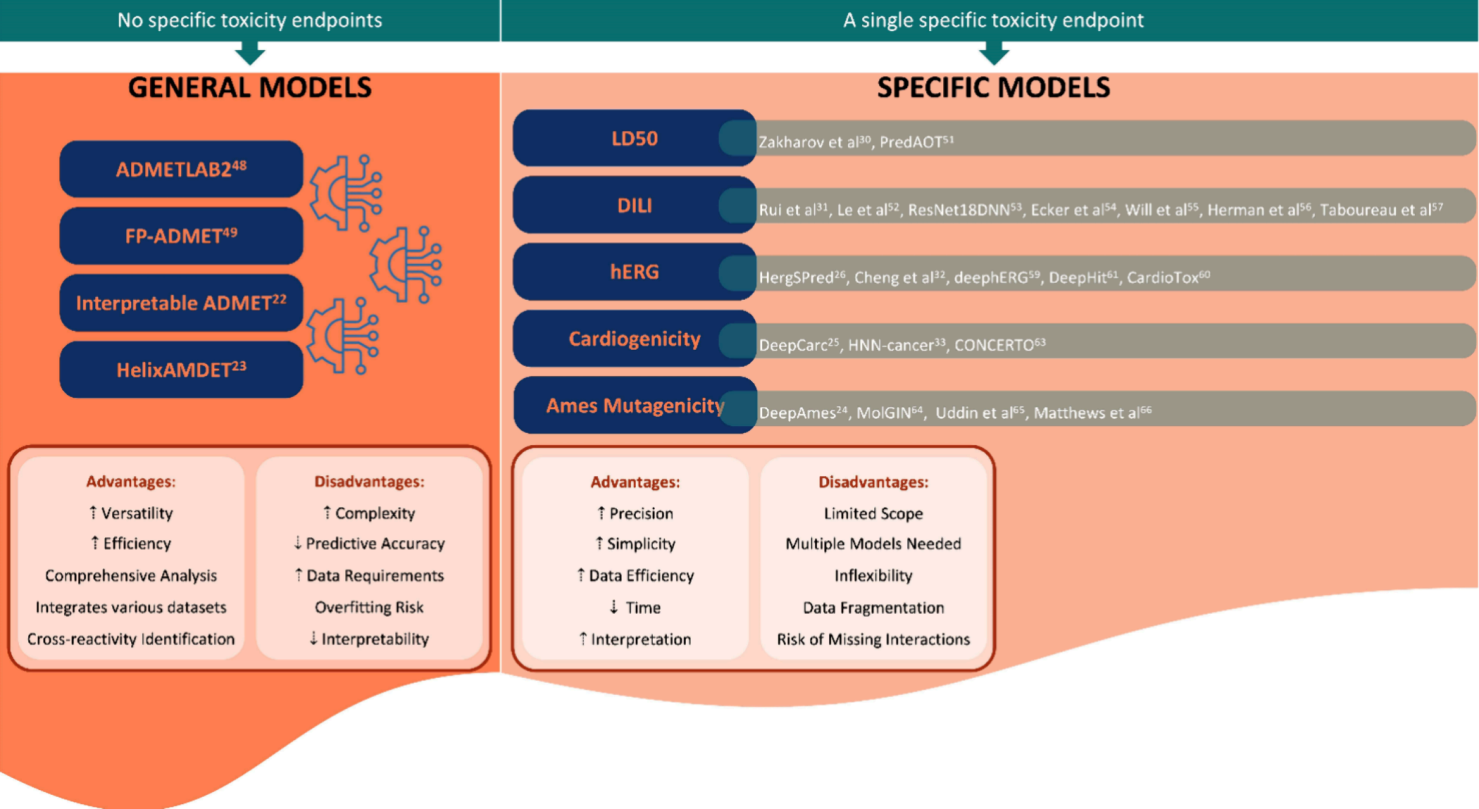
General and specific models in toxicity prediction, encompassing the model’s generality or specificity regarding predicted toxicity end points (lethal dose 50% (LD50), drug-induced liver injury (DILI), human ether-a-go-go-related gene (hERG inhibition), carcinogenesis, and Ames mutagenicity), along with its advantages and disadvantages.

ADMETLAB2,^48^ FP-ADMET,^49^ Interpretable ADMET,^22^ and HelixAMDET^23^ are general methods used to predict several ADMET end points. Each one with them on type of architecture, they all show advantages in terms of time efficiency, providing comprehensive and holistic view of multiple aspects of drugs and integration with systems pharmacology. With regard to particular end points, certain end points, such as LD50, DILI, hERG, carcinogenicity, and Ames mutagenicity, are of utmost significance (Figure 3).

#### 3.2.1 LD50

As a significant advancement in toxicology research, Jain et al. (2021) addressed the challenge of predicting compound toxicity across various exposure routes and species. They developed multitask ensemble models specifically designed for toxicity prediction, covering 59 toxicity end points across different species and exposure scenario ends.^30^ This study demonstrated the higher performance of this multitask ensemble model compared with single-task models, thus confirming the results of Sosnin et al.^50^ Moreover, it contributes to understanding the safety across a variety of species and different exposure scenarios, thus addressing another limitation of interspecies variability. A key strength of Jain et al.’s study is the utilization of a large, publicly available data set, which bolsters the study’s transparency and allows greater reproducibility. This openness invites the broader research community to validate and expand their findings, fostering a collaborative approach to advancing toxicological science. However, the study’s reliance on a single data source while ensuring consistency may also restrict the diversity of the data set, potentially overlooking various perspectives or findings. This could potentially narrow the applicability of the model, particularly for fewer common species. Furthermore, the presumption that a finite set of mechanisms can explain chemical toxicity across various species might not fully encompass the intricate biological responses to chemical exposure, highlighting a potential area for further investigation. The recently released PredAOT^51^ was characterized by the authors as a valuable tool for the rapid and precise prediction of acute oral toxicity of small compounds in mice and rats. PredAOT employs a two-step approach: initially classifying compounds into toxic or non/less toxic categories, followed by specific LD50 predictions via downstream regressors tailored to toxicity classification. Its ability to predict acute toxicity in both mice and rats, thereby achieving or exceeding the performance of existing methodologies, is a significant achievement. The focus on data balance in this research tackles a crucial issue that is frequently neglected: the risk of bias, which leads to more dependable predictions. The model’s user-friendly interface further increases its accessibility and broadens its utility within the scientific community. However, a common drawback of these approaches is their dependence on information from a single database, which potentially limits the data diversity and, consequently, the generalizability of the results. Future studies should endeavor to include data from various sources to strengthen the robustness and completeness of the data sets. Making these diverse data sets openly accessible would allow the scientific community to collaboratively refine our understanding of species-specific toxic responses and advance toxicological research.

#### 3.2.2 DILI

The landscape of DILI prediction has been notably enriched by recent advancements, featuring a variety of models that propose innovative solutions to this complex problem. Among these, the employment of gene expression data through deep learning (DL) models,^31^ including Convolutional Neural Network (CNN) and Natural Language Processing (NLP)-inspired frameworks,^52^ represents a promising strategy for accurately forecasting hepatotoxicity. The inclusion of gene expression data shows promise in predicting this toxicity end point and potentially predicting other end points. Another advantage is that they used a data set known to be a good proxy to predict hepatotoxicity in humans. However, the reduced availability of data is one of the main limitations, since only 87 compounds were associated with the 988 samples used. This scarcity of data underscores a critical challenge in biomedical research: the pressing need for broader data availability to propel healthcare and research. On the recommendation front, harnessing embedding techniques and molecular fingerprinting for enhancing model efficiency is an essential aspect of toxicological prediction. The application of ResNet18DNN, introduced by Chen et al., along with methods for vectorizing molecular-structure images, has emerged as an innovative beacon of innovatione.^53^ By carefully sourcing data from an array of literature and databases, the ResNet18DNN significantly mitigates the issue of data set diversity and size, a worthy effort that sets a benchmark for future studies. However, the integration of diverse molecular embedding techniques, while advantageous, inevitably complicates the interpretability and usability of the model. A more systematic approach for optimizing these compound description methods is imperative to streamline the model without compromising its robustness. Moreover, there is a critical need to investigate the development of personalized prediction models for liver injury, recognizing the significant variability in drug or compound effects due to a genetic predisposition to DILI. Furthermore, there is a need for personalized DILI prediction models because they acknowledge the vast interindividual variability influenced by genetic predispositions. This variability is not merely a footnote but a central consideration in the development of predictive models. Füzi et al.’s^54^ introduction of a systems biology approach, utilizing target, interactome, and pathway profiles, offers a fresh lens through which to view hepatotoxicity mechanisms. This approach is not just another method but also a potential revolution in understanding the biological intricacies of DILI. Similarly, an innovative method that combines predicted off-targets with interpretable molecule descriptors to discern DILI potential presents a forward-thinking strategy. The promise of incorporating gene expression data into future methodologies is particularly exciting, heralding a new era of predictive modeling that embraces the complexity of biological systems.^55^ Two other approaches with the potential to contribute to DILI prediction were released in 2023: one based on in vivo studies that capture mechanistic and phenotypic DILI information more reliably and accurately than other data sets, using in vivo liver histopathology nomenclature to provide a more informative and reliable data set for ML algorithms,^56^ and another using cell painting and transcriptomic data on compounds. This demonstrates the advantages of high-content imaging using transcriptomics data.^57^ These studies highlight the importance of combining different modalities for comprehensive toxicity assessments in drug discovery, such as integration of chemical and biological data, as mentioned by Liu et al.^58^ This multifaceted approach has the potential to lead to more robust predictive models, ultimately improving the safety and efficacy of new therapeutics. The journey toward safer therapeutics is both challenging and exciting, with much ground yet to be covered.

#### 3.2.3 hERG Cardiotoxicity

Similar to other end points, hERG cardiotoxicity has been used to develop accurate prediction models. Beginning in 2019, a multitask DNN (MT DNN)-based model, deephERG,^59^ was published to predict the hERG blocker activity for small molecules. The superior performance of deephERG over traditional machine learning methods, including Naïve Bayes (NB), Support Vector Machine (SVM), Random Forest (RF), Graph Convolutional Network (GCN), and single-task DNNs, underscores the potential of Multitask Learning (MTL). By facilitating knowledge sharing across tasks, multitask models reveal patterns obscured in isolated task analyses, offering a richer understanding of hERG blocker activity prediction. The deephERG model exemplifies the utility of MTL, suggesting a promising avenue for future exploration aimed at bolstering predictive accuracy. Subsequently, CardioTox, a method for efficiently aggregating information derived from models that rely on diverse chemical representations, was proposed by Karim et al.^60^ The authors referenced DeepHIT, a framework developed by Ryu et al.,^61^ and incorporated a DNN and GCN to rationalize the development of CardioTox. This decision stems from the challenges associated with aggregating the extracted information and addressing the low performance observed in various metrics for DeepHIT. Another finding from researchers was the potential to improve performance across a diverse set of metrics by leveraging high-level (global characteristics, such as 2D and 3D properties) physicochemical, low-level (detailed representations of molecular structures) fingerprints, Simplified Molecular Input Line Entry System (SMILES) embedding vectors, and fingerprint embedding vectors as meta-features for the meta-ensemble. This methodological diversity signifies a shift toward more holistic assessments, blending various data types, and computational techniques to overcome the challenges of cardiotoxicity evaluation. An alternative approach to predict compounds with potential hERG-induced cardiotoxicity was proposed in 2022 by Shan et al.^32^ They released an unnamed Directed Message-Passing Neural Network (D-MPNN)-based model for predicting hERG channel blockers. Despite achieving a comparable performance to CardioTox, this model underscores an ongoing challenge in the field: the dire need for extensive, high-quality, and unbiased data sets. The recurrent emphasis on data quality and quantity reveals a fundamental bottleneck in advancing predictive models, which is a limitation that cannot be overlooked. In the same year, CardioTox was outperformed by the HergSPred^26^ classification method for hERG blockers and nonblockers. Using an ensemble approach that leverages DNN, RF, and extreme Gradient Boosting (XGBoost),^62^ they also examined the contribution of each input fingerprint to the prediction output, thus gaining insight into warning substructures that might be indicative of the cardiotoxicity of the input compound. This analytical journey through recent methodologies underscores critical dialogue in the field of cardiotoxicity prediction. Although computational models have advanced significantly, the integration of diverse computational strategies and the amplification of data quality are pivotal challenges. Moreover, the exploration of ensemble and multitask models reveals a path toward more nuanced and accurate predictions, yet the journey is far from complete. The continuous refinement of these models coupled with an unwavering commitment to data enhancement heralds a future in which predictive accuracy can substantially reduce the risk of hERG-induced cardiotoxicity in drug development.

#### 3.2.4 Carcinogenicity

Considering the notable advantages of DL methods in predicting drug toxicity, DL-based models have been used to predict carcinogenicity. These advanced models, with their complex architectures and ability to process intricate data sets, provide a glimpse into the future of the early detection and evaluation of the carcinogenic potential of compounds. Upon conducting a thorough analysis of recent methodologies, a critical evaluation revealed the progress made and the challenges that still remain. DeepCarc, proposed by Li et al.^25^ appears to be a potential early detection tool for carcinogenicity assessment and screening for the carcinogenic potential of compounds from both DrugTax and Tox21. The authors recalled one of the major challenges of AI models is explainability. They focused on the limitations inherent to uniform manifold approximation and projection techniques to enhance the transparency of the model. However, exploration of misclassified carcinogens underscores a pivotal issue: the occurrence of false negatives, which remains a significant concern in practical applications. This highlights the essential role of complementary methods, such as high-throughput in vitro toxicity assays, in refining prediction accuracy and reducing the risk of overlooking carcinogenic compounds. Another method, considered first-in-class upon release in 2022, takes advantage of a hybrid DNN/CNN architecture to develop three types of ML models: binary classification models to predict whether a chemical is carcinogenic or noncarcinogenic; multiclass classification models to predict the severity of chemical carcinogenicity; and regression models to predict the median toxic dose of chemicals.^33^ Limbu et al. acknowledged some limitations in the prediction of carcinogenicity using their model, such as the absence of a large dose-dependent chronic in vitro and in vivo carcinogen data set to train the model, and the need for further refinement. This reiterates the crucial aspect of enhancing data availability and sharing. Therefore, to overcome the data-size challenge, CONCERTO by Fradkin et al.^63^ was developed as a multiround pretraining methodology that uses mutagenic data to enhance the accuracy of the carcinogenicity task. Moreover, this combined graph transformer with a molecular fingerprint representation to predict carcinogenicity based on molecular structure outperforms the previous state-of-the-art methods and addresses another limitation by further analyzing model interpretability. This advancement signals a shift toward models that not only predict with higher accuracy but also provide insights into their decision-making processes, enhancing the understanding of how molecular structures relate to carcinogenic potential. The path forward for DL in carcinogenicity prediction is marked by a dual emphasis on enhancing model performance and ensuring model explainability. Techniques, such as data augmentation and transfer learning, have emerged as promising strategies to circumvent data scarcity, enabling models to learn from limited data sets more effectively. However, the pursuit of explainable features remains paramount, ensuring that future methodologies can be fully understood and trusted by researchers and regulators.

#### 3.2.5 Ames Mutagenicity

Finally, the Ames Mutagenicity assessment saw a notable development in 2020 with the introduction of MolGIN by Peng et al.^64^ using a promising type of Graph Neural Network (GNN): the Graph Isomorphism Network (GIN). MolGIN exploits the bond features and differences in the influence of atom neighbors to predict the ADMET properties. MolGIN showed that it significantly boosted the prediction performance of the GIN and outperformed the baseline models. Despite these advancements, MolGIN’s interpretability remains a challenge, particularly in identifying specific substructures within molecules that significantly contribute to prediction outcomes, highlighting a gap in our understanding of the molecular underpinnings of mutagenicity. In the following year, Kumar et al.^65^ got to the conclusion that the idea of “the deeper the network, the better the result” does not always hold. Despite their emphasis on the need for an optimized depth in DNNs and the fact that going beyond that depth does not necessarily lead to improved results, two different approaches have emerged for predicting mutagenicity. DeepAmes^24^ has shown promise as an essential tool in regulatory reviews, aiding in the assessment of the potential mutagenicity of drugs, drug impurities, food additives, and various environmental and industrial chemicals. Despite these efforts, the explainability of DeepAmes still requires improvement. Given the importance of interpretability, which is one of the most significant model constraints, it should not be overlooked and warrants consideration in the future. The second addresses the fact that some molecules become mutagenic only after metabolic activation, which has not been fully considered in traditional models. The Multiple Instance Learning (MIL) method developed by Feeney et al.^66^ suggested its potential to improve prediction accuracy, particularly for molecules with complex metabolic pathways. Advocacy for open-source code and data sharing has emerged as a consistent theme across these developments, emphasizing the importance of transparency and collaboration in advancing the field. Furthermore, recent explorations of multitask learning and transfer learning have revealed their potential to significantly enhance prediction models. Specifically, neural networks organized around mechanistic task groupings have demonstrated superior performance compared to both ungrouped neural networks and single-task models.^67^ This success underscores the utility of multitask learning frameworks and transfer learning in leveraging shared knowledge across related tasks, thereby offering a promising avenue for future research on mutagenicity prediction.

Considering the emphasis on these toxicity end points, other studies have shown progress over the years. NOAEL and LOAEL, which are challenging to model, were thoroughly investigated in 2021, leading to a confirmed modeling strategy that uses in silico models to assess the repeated-dose toxicity of chemicals.^68^ The evolution continues with new approaches and architectures in the realm of molecular and atomic representation for toxicity prediction, revolutionizing the field with innovative ML models. Traditional GNNs capture complex atomic interactions using only atomic coordinates and types. Shifting toward 3D GNNs, particularly employing a Message-Passing Neural Network (MPNN) framework based on molecular geometry, allows for refining high-dimensional atomic representations crucial for predicting specific molecular properties.^69^ Notably, the incorporation of physical inductive biases into these models ensures success through constraints on the input space or embedding biases within the model mechanics.^70^ Among these cutting-edge models, the Equivariant Graph Neural Network (EGNN) TorchMD-NET stands out, showing promising potential for generating precise atomic and molecular structure representations for advancing QSAR modeling in toxicity prediction.^71^ This trajectory provides deeper insights into the development of robust and reliable predictive models for toxicity assessments. Ongoing advancements in ML models, including innovative and powerful approaches, offer promise for revolutionizing toxicity predictions. Nevertheless, addressing significant challenges, such as data quality, availability, balance, interpretability, and fostering interdisciplinary efforts remains crucial.

Animal models are often inaccurate for predicting human toxicity, making it difficult to differentiate toxicity in in vitro models.^13^ Therefore, new strategies are required to improve the ability to predict human outcomes. By supplementing general AI-based models for predicting toxicity, specific AI-based models offer added value. A comprehensive overview of existing approaches is presented in Table 1.

**Table 1:** Summary of the Performance of State-of-the-Art Models for Toxicity Prediction Grouped by Toxicity End Points^a^

end point	authors	name	method	data sets	data type	data set size/compounds	data split	RMSE	R^2^	PCC	SCC	AUC	Acc	MCC	Sen
LD50	(30)	-	Multitask Ensemble (DNN, CNN, GCN, RF)	ChemIDplus	drug-like space	80 081	random split	0.650	0.570						
	(49)	FP-ADMET	RF	4 (including EPA)	drug-like space	11 363	random/5-fold cross-validation					0.810	0.680		
	(48)	ADMETLAB 2.0	MGA	ChEMBL, PubChem, OCHEM, literature	drug-like space	3039	random split ten times					0.853	0.778	0.549	0.793
	(22)	Interpretable ADMET	GCN/GAT	ChEMBL, PubChem, DrugBank, literature	drug-like space	7334	random split five times	0.106	0.575						
	(23)	HelixADMET	GNN	ChemIDplus	drug-like space	3757	random split					0.808			
	(51)	PredAOT	RF	OCHEM	drug-like space	6226 for mice; 6238 for rats		0.532	0.307	0.798	0.764	0.744		0.493	
Liver Toxicity	(31)		DL, SVM	ArrayExpress	drug-like space	988	random split					0.989	0.971	0.942	0.974
	(52)		CNN	5	drug-like space	1919	random split 30 times					0.960	0.890	0.800	
	(49)	FP-ADMET	RF	Several from literature	drug-like space	2478	random/5-fold cross validation					0.880	0.790		
	(48)	ADMETLAB 2.0	MGA	ChEMBL, PubChem, OCHEM, literature	drug-like space	1339	random split ten times					0.924	0.894	0.793	0.958
	(22)	Interpretable ADMET	GCN/GAT	ChEMBL, PubChem, DrugBank, literature	drug-like space	644	random split five times					0.718	0.723	0.450	0.628
	(23)	HelixADMET	GNN	Mulliner et al., 2016	drug-like space	1904	random split					0.808			
	(53)	ResNet18DNN	DNN	7	drug-like space	1446	random split					0.958	0.976		
	(54)	-	RF	8 (including ChEMBL and DrugBank)	drug-like space	452	random split/5-fold cross validation						0.766		0.730
	(55)	-	Consensus (KNN, SVM, RF, NB, ANN, LR)	FDA, literature	drug-like space	603	random split/10-fold cross validation					0.880	0.810		0.730
	(56)	-	RF, SVM and LogR	3	drug-like space	430	5-fold stratified group cross-validation ten times					0.640–0.690			
	(57)	-	RF, SVM and ElasticNet	2 (including eTox)	drug-like space	537	5-fold stratified group cross-validation two times						0.645- 0.739		
hERG Cardiotoxicity	(59)	deephERG	MT-DNN	4 (including ChEMBL)	drug-like space	7889						0.967			0.926
	(61)	DeepHIT	DNN/GCN	6 (including BindingDB, ChEMBL, and in-house)	drug-like space	14 440						0.773	0.476	0.833	0.643
	(60)	CardioTox net	MT-DNN (DNN, GCN, CNN)	6 (including DeepHIT, ChEMBL, BindingDB)	drug-like space	12 620	10-fold cross validation split					0.930	0.860	0.720	
	(49)	FP-ADMET	RF	ChEMBL, Siramshetty et al., 2019, Cai et al. 2019	drug-like space	7889	random/5-fold cross validation					0.880	0.800		
	(48)	ADMETLAB 2.0	MGA	ChEMBL, PubChem, OCHEM, literature	drug-like space	1332	random split ten times					0.943	0.889	0.778	0.909
	(22)	Interpretable ADMET	GCN/GAT	ChEMBL, PubChem, DrugBank, literature	drug-like space	8672	random split five times					0.784	0.919	0.612	0.603
	(23)	HelixADMET	GNN	ChemIDplus, PubChem, literature	drug-like space	1747	random split					0.909			
	(32)	-	D-MPNN	5 (including Cai)	drug-like space	14 409	random split/5-fold cross validation					0.956			
	(26)	HergSPred	Ensemble (DNN, RF, XGBoost)	5 (including ChEMBL)	drug-like space	12 850						0.908	0.840	0.681	0.824
Carcinogenicity	(25)	DeepCarc	DNN	NCTRlcdb	drug-like space	863	kennard-stone (ks) algorithm split					0.776	0.754		
	(49)	FP-ADMET	RF	Bercu et al., 2010, Zhang et al., 2017	drug-like space	1003	random/5-fold cross validation					0.750	0.680		
	(48)	ADMETLAB 2.0	MGA	ChEMBL, PubChem, OCHEM, literature	drug-like space	1982	random split ten times					0.778	0.731	0.476	0.843
	(22)	Interpretable ADMET	GCN/GAT	ChEMBL, PubChem, DrugBank, literature	drug-like space	1146	random split five times					0.690	0.761	0.387	0.593
	(23)	HelixADMET	GNN	PubChem	drug-like space	3206	random split					0.836			
	(33)	HNN-Cancer	DNN/CNN	6 (including DSSTOX)	broad chemical space	7994	5-fold cross validation					0.806	0.743		
	(63)	CONCERTO	GNN	CPDB and CCRIS	broad chemical space	6540	3-fold cross validation					0.730			
Ames Mutagenicity	(64)	MolGIN	GIN	7 (including Tox21)	drug-like space	7619	5-fold cross validation					0.918	0.839		
	(65)		DNN, SVM, KNN, RF	5	drug-like space	4053	stratified 10-fold cross validation					0.894	0.838		
	(49)	FP-ADMET	RF	Sushko et al., 2011, Xu et al., 2012	drug-like space	7950	random/5-fold cross validation					0.870	0.790		
	(48)	ADMETLAB 2.0	MGA	ChEMBL, PubChem, OCHEM, literature	drug-like space	3304	random split ten times					0.902	0.807	0.606	0.865
	(22)	Interpretable ADMET	GCN/GAT	ChEMBL, PubChem, DrugBank, literature	drug-like space	7387	random split five times					0.842	0.843	0.682	0.832
	(23)	HelixADMET	GNN	Xu et al., 2012	drug-like space	4147	random split					0.909			
	(24)	DeepAmes	Ensemble (KNN, LR, RF, SVM, XGBoost, DNN)	DGM/NIHS	broad chemical space	11 569	grid search with a bootstrap aggregating strategy					0.740	0.840	0.380	0.470
	(66)		MIL	OECD QSAR Toolbox, literature	aromatic amine chemical space	6505	stratified split						0.778		

a Abbreviations: Acc: Accuracy^b^, ANN: Artificial Neural Network, AUC: Area Under the ROC Curve^c^, CNN: Convolutional Neural Network, D-MPNN: Directed Message-Passing Neural Network, DL: Deep Learning, DNN: Deep Neural Network, GAT: Graph Attention Networks, GCN: Graph Convolutional Network, GIN: Graph Isomorphism Network, GNN: Graph Neural Network, KNN: K-Nearest Neighbors, LogR: Logistic Regression, LR: Linear Regression, MCC: Matthews Correlation Coefficient^d^, MGA: Multitask Graph-Attention, MIL: Multiple Instance Learning., MT-DNN: Multitask DNN, NB: Naive Bayes, PCC: Pearson Correlation Coefficient^e^, R^2^: Coefficient of Determination^f^, RF: Random Forest, RMSE: Root Mean Square Error^g^, SCC: Spearman Correlation Coefficient^h^, Sen: Sensitivity^I^, SVM: Support Vector Machine, XGBoost: eXtreme Gradient Boosting.

b Accuracy measures the overall correctness of the predictions made by the model. Accuracy is calculated by dividing the number of correct predictions by the total number of predictions.^72^ It ranges between 0 and 1, where higher accuracy indicates better model performance.

c The AUC is used to represent the area under a curve in a graphical representation, typically used in binary classification problems.^73^ An AUC of 1 indicates good model performance.

d The MCC considers true positive, true negative, false positive and false negative values.^73^ A value of 1 indicates a perfect prediction 0 suggests a random prediction and −1 represents a complete disagreement between predictions and observations.

e The PCC is a measure of the linear correlation between two variables indicating the strength and direction of their relationship. It ranges from −1 (perfect negative correlation) to 1 (perfect positive correlation) with 0 indicating no correlation.^74^

f R^2^ represents the proportion of variance in the dependent variable that is predictable from the independent variables.^75^ An R^2^ value of 0 indicates that the model does not explain any of the variability in the dependent variable and an R^2^ value of 1 indicates that the model perfectly explains the variability in the dependent variable.

g The RMSE is calculated by taking the square root of the average of the squared differences between the predicted and actual values.^76^ Lower RMSE values indicate a better model performance.

h SCC is a nonparametric measure of the statistical dependence between two variables. It ranges from −1 (perfect negative monotonic relationship) to 1 (perfect positive monotonic relationship) with 0 indicating no monotonic relationship.^74^

I Sensitivity measures the ability of a model to correctly identify positive instances out of the total actual positive instances in a data set.^72^ It ranges from 0 to 1 where 0 indicates that the model fails to correctly identify any positive instance.

## 4 Current Paradigm for Binding Affinity Prediction

Binding affinity is a measure of the interaction strength and is usually expressed as IC_50_, *K*_i_, or *K*_d_.^77^ Therefore, DTBA is used to describe drug efficacy and to quantify the potency of a drug and the strength of binding between a drug and its target. As previously mentioned, DTBA prediction is a fundamental step in Virtual Screening (VS) and de novo drug design. AI techniques have been implemented because of computational evolution.^78−80^ ML can address this issue as a regression or classification problem.^79,81^ In the case of classification, algorithms are binary classifiers that aim to label unknown compounds as active or inactive based on a threshold defined a priori that establishes the minimum value for ligand activity^81^ or through the description of well-known interactions.^82,83^ For instance, when using the ChEMBL database, a common threshold of bioactivity is an IC_50_ of 10 μM, which corresponds to a pIC_50_ of 5, although refinement of this value based on the data distribution has been proposed.^84^ Affinities below the threshold represent active ligands, whereas those above the threshold represent inactive ligands. This is easy to comprehend considering that a higher IC_50_ value indicates that a higher concentration of the drug is necessary to induce inhibition, and thereby, a lower affinity exists. In contrast, regression models predict a continuous value of binding affinity based on known samples; this value was defined.^80,81^

### 4.1 Enhancing Drug Safety through DTBA Prediction and Off-Target Effect Analysis

Predicting drug toxicity involves a deep understanding of the molecular interactions between drugs and target proteins, including potential adverse effects on the human body. The key to this is the binding affinity of drugs to their targets, as reflected by DTBA. This affinity is crucial for anticipating drug-target interactions, including both on-target (therapeutic) and off-target (often toxic) interactions, which are integral to assessing drug toxicity. On-target toxicity, a scenario in which interaction with the intended target leads to adverse effects, underscores the dual nature of drug interactions; they can be both therapeutic and toxic.^85^ Enhancing DTBA prediction not only improves the accuracy of toxicity forecasts but also aids in identifying unintended off-target interactions. The integration of computational methods in forecasting drug toxicity is instrumental in leveraging DTBA predictions to enhance model insights into potential interactions, thereby augmenting the predictive accuracy of toxicity models. Consequently, the integration of computational techniques bolsters the predictive power of models and plays a significant role in efficacy and safety evaluations during drug development and optimization.^86,87^

Furthermore, detecting off-target effects through the prediction of the DTBA encompasses a range of computational and experimental strategies. Computational tools leverage diverse approaches, including target-centric methods such as structure-based systems biology, protein–ligand docking, and data fusion. These techniques aim to anticipate off-target interactions, which are normally characterized by a lower affinity compared to the intended pharmacological targets. Although experimental evidence for off-target interaction predictions is limited, these computational approaches show promise for identifying off-target effects and guiding further experimental studies.^88^ Moreover, unraveling the mechanisms underlying drug toxicity, particularly the roles of targets and pathways, is frequently neglected when evaluating pharmaceutical candidates. The availability of extensive data on adverse outcome pathways is essential to construct reliable predictive models.^58^ Harnessing drug target binding affinity significantly improves the precision of drug toxicity predictions, shedding light on the complex interactions within biological networks.^89^

### 4.2 DTBA as Features and its Data Sources

Drug target binding affinity plays a crucial role in predicting drug toxicity as it determines the specificity of on-target binding, required dosage and concentration, off-target effects, duration of effect, therapeutic window, and potential drug interactions. High binding affinity to an on-target protein is more likely to result in a lower effective dose, thereby reducing the risk of side effects. Conversely, nonspecific binding to multiple targets may lead to increased toxicity, unintended interactions, and off-target effects. Understanding binding affinity is essential for predicting off-target and potential drug interactions, particularly when off-target proteins are critical for physiological functions. Additionally, a high binding affinity to on-target proteins may lead to a narrow therapeutic window, requiring precise dosing and monitoring to avoid toxicity. Personalized medical approaches that consider individual differences in protein expression and function are important for predicting drug toxicity in different individuals. Furthermore, it signifies a paradigm shift toward a more complex understanding of drug behavior at the molecular level, allowing for more informed decision-making in drug development, ultimately leading to improved patient care and outcomes. Recently developed predictors have been implemented using supervised and semisupervised learning approaches.^90−92^ Furthermore, they involve graphs,^93,94^ ML algorithms,^90,95^ DL,^77,96,97^ or a combination of both.^95,98^ In general, these models can be classified as feature- or similarity-based;^96,99^ however, ensemble techniques can include both procedures.^79^ A feature-based method attributes a feature vector composed of descriptors that characterize both the ligand and protein. Commonly, features are extracted separately for the drug and target and subsequently concatenated to produce an interaction feature vector.^79,96^ Similarity methods infer drug activity, considering that similar ligands interact with similar targets and reciprocally that similar proteins are targeted by similar ligands.^79,96^ This correlation is known as the “guilt by association” rule.^100^ The data used in these methods are generally retrieved from public databases or data sets. Databases such as DrugBank^101^ and the Kyoto Encyclopedia of Genes and Genomes Drug (KEGGDRUG)^102^ provide information regarding drugs and targets that can be used in DTI studies. However, only a few databases provide information on binding affinity. These included ChEMBL,^103^ Binding Database (BindingDB),^104^ BindingMother of All Databases (BindingMOAD),^105^ STITCH,^106^ PubChem, and Protein Database (PDB) Bind (PDBBind).^107^

ChEMBL is a database that is used in experimental assays. It provides a detailed description of the results obtained, assay conditions, and elements involved.^103^ All information was retrieved from the literature, curated by humans, and properly standardized.^108^ It contains 2 582 500 experimental IC_50_ values. BindingDB is composed of 1 652 880 IC_50_ scores experimentally determined from protein-small molecule complexes, and it includes data extracted from PubChem and ChEMBL.^104^ BindingMOAD is a subset of PDB that contains high-resolution structures composed of ligands of interest for which affinity data have been retrieved from the literature.^105,109^ Among the 12 098 binding affinity values, 4182 were indicative of IC_50_. STITCH^106^ is an interaction database that includes both drug-target and protein–protein pairs. It aims to centralize information from multiple databases, including information, in addition to binding affinity. For example, it is suitable for conducting network analysis. It is a repository of 9 600 000 proteins and 430 000 ligands. PubChem is a public database of the National Institutes of Health (NIH) that provides a detailed description of chemical compounds, mainly small molecules, including their physicochemical and bioactivity properties, toxicity, safety, and patents.^110^ It contains 271 M bioactivity scores. Finally, PDBBind reports the experimentally determined binding affinities of PDB complexes.^107^

Thafar et al. summarized the most common data sets used to assess bioactivity.^79^ However, the number of benchmark data sets is lower than the number of databases. Four popular data sets were used to develop state-of-the-art models: Yamanishi data set,^111^ Davis,^112^ Kinase Inhibitor BioActivity (KIBA),^113^ and Metz.^114^ Yamanishi et al. described protein–ligand interactions involving four protein families: nuclear receptors, G Protein-Coupled Receptors (GPCRs), ion channels, and enzymes. Each drug-target complex is labeled as interacting or noninteracting; therefore, it is valuable for developing classification models.^111^ The remaining data sets are useful for both the regression and classification models because they quantify the binding affinity of each complex. All three refer to the interactions in which the targets belong to the kinase family. The KIBA data set scores the binding affinity in terms of the KIBA score, which is a measurement obtained from *K*_d_, *K*_i_, and IC_50_ values. A higher KIBA score corresponds to lower affinity.^113^ It describes 118 254 interactions between 2116 drugs and 229 proteins, with activity values ranging from 9 to 16 on a −log_10_ scale.^90,113^ Davis reported approximately 30 056 interactions involving 68 ligands and 442 targets, expressing affinity in the form of −log_10_(*K*_d_), whose values varied from 5 to 9.5,^90,113^ whereas Metz et al. reported a *K*_i_ of 35 259 between 1421 compounds and 156 kinases.^114^ Metz activity values on a log_10_ scale ranged from 4 to 9.^90^

### 4.3 AI-Based Models

Traditional methods for predicting DTI face financial and technical constraints, prompting a shift toward more efficient computational strategies. Key computational approaches include ligand-based methods, docking simulations, chemo-genomic approaches, text mining, and ML/DL methods. Many existing approaches treat DTI prediction as a binary on–off relationship, struggling to differentiate between true negatives and instances where the absence of interaction is due to experimental limitations.^79^ Recent studies have focused on predicting the DTBA to gauge the strength of the interaction. ML and DL methods have been employed to overcome the shortcomings of binary DTI prediction, and principal studies in this area have dealt with binding affinity data expressed as IC_50_, *K*_i_, or *K*_d_.^115,116^ All the approaches mentioned herein are relevant for resolving the DTBA problem, contributing to the development of more rigorous pipelines and the evolution of model architectures. The prediction of DTI binding affinity has been interpreted in two ways: as a classification task, which involves labeling a drug-target pair as active (1) or inactive (0), or as a regression task, which aims to predict a continuous score of binding affinity. Moreover, some methods have attempted to forecast only a single metric or more than one. Because regression models are more informative than simple classifications, a review of the existing regression models for DTBA prediction based on sequence-derived information will be conducted to provide a detailed understanding of the current landscape.

KronRLS^116^ was the most effective similarity-based technique. This method was reported in 2015 using PubChem clustering software to retrieve ligand similarity and the Smith-Waterman algorithm to calculate the distance between targets. KronRLS, uses the Kronecker product to predict DTBA. However, it was less effective in handling novel drugs than the SimBoost^90^ algorithm proposed in 2017. The (SimBoost) Gradient Boosting Machine (GBM) approach was proposed to predict the DTBA scores. It outperformed KronRLS on all three data sets, displaying superior Root Mean Square Error (RMSE) and Concordance Index (CI) values. SimBoost’s feature extraction involves the construction of a similarity matrix for compounds and targets, and it performs better than KronRLS in benchmarking tests. By not utilizing a similarity matrix, PADME,^96^ a DNN predictor developed in 2018, addresses the limitations of KronRLS and SimBoost, making it more computationally efficient. PADME outperformed both KronRLS and SimBoost, demonstrating the value of feature-based DNN approaches. While KronRLS was effective under certain conditions, subsequent models, such as SimBoost and PADME, demonstrated improved performance, highlighting the continuous advancement in the field.

However, acknowledging DeepDTA^77^ is required because it emerged as a pioneer among DL-based DTBA prediction models in 2018. Operating as a text-based DNN, it employs a 1D representation for both drugs and targets, with SMILES serving as the input data for drugs. The authors verified that the use of a separate CNN to represent drugs and targets was favorable for executing more accurate predictions. It was concluded that the application of DL to sequence-derived information decreased the error associated with the prediction of DTBA as the size of the data set increased, making it more appropriate for addressing big data. The following year, WideDTA^117^ was introduced as an updated version of DeepDTA. Both methods employ text-based information to describe the proteins and ligands. However, WideDTA constructed words using protein sequences, ligand SMILES, protein motifs, domains, and the maximum common substructures of ligands, whereas DeepDTA only extracted characters from the protein sequences and ligand SMILES. Researchers have demonstrated that this improved CNN-based method surpasses DeepDTA.^117^

In 2019, the potential of graph attention networks was evaluated using the MT-DT,^118^ IVPGAN,^119^ and GANsDTA^92^ methods. A previous study has presented a self-attention approach that learns the structure of molecules from their sequences. SMILES, obtained from PubChem, was used to structurally represent the molecules, protein sequences, and ligands. Subsequently, this new representation is transferred to a DNN that predicts protein–ligand interactions. MT-DTI outperformed KronRLS and DeepDTA in terms of RMSE, rm^2^, and CI. Finally, IVPGAN and GANsDTA use Generative Adversarial Networks (GANs) to solve the DTBA problem. The latter approach takes advantage of these types of networks for sequence-based feature extraction and proposes two GANs: one to characterize proteins, and the other to characterize ligands from SMILES. Predictions were made using a CNN. Therefore, this method was performed in a manner similar to that used for DeepDTA.^92^

The potential of GNNs, molecular graphs, and structural information for improving the performance of DTBA prediction models was demonstrated in 2020. GraphDTA^94^ demonstrated that GNNs were more promising than simpler CNN approaches as they successfully exceeded DeepDTA. In the same year, DGraphDTA^93^ used two graphs to represent proteins and ligands based on structural information, achieving superior performance compared to KronRLS, SimBoost, DeepDTA, GraphDTA, and WideDTA. This model used a contact map to introduce 2D representations of the 3D structures of proteins and showed better performance than other models. A Multi-Objective Neural Network (MONN)^120^ was proposed to predict the binding affinity using protein amino acid sequences and a graph-based representation of ligands, outperforming KronRLS, SimBoost, DeepDTA, GraphDTA, and WideDTA.

The chronological evolution from traditional similarity-based techniques to sophisticated DNNs and GNNs underscores the continuous progress in enhancing DTBA prediction and gaining insights into drug-target interactions, toxicity prediction, and off-target effects. Nonetheless, some of the most recent studies released in 2022 include Affinity2Vec,^81^ DeepMHADTA,^115^ and WGNN-DTA.^121^ Affinity2Vec is a graph-based model that exclusively integrates sequence-derived information, thereby dispensing structural information. This approach proposes the construction of drug and target embeddings that are subsequently utilized in a graph. DeepMHADTA suggests a Multi Head Attention (MHA) mechanism-based model using SMILES, target sequences, as well as the secondary structure of proteins and structural fingerprints of ligands. The authors concluded that their approach has the advantage of using a more complex Artificial Neural Network (ANN) than state-of-the-art methods, applying NLP to extract protein sequence features, and further considering their 2D structure, WGNN-DTA, an improvement of DGraphDTA, introduced a Weighted Graph Neural Network (WGNN) for DTBA prediction, considering edge weights based on the probability of interaction for protein residues and molecular graphs for ligands. These models represent ongoing innovations in DTBA prediction, incorporating diverse approaches for improved accuracy and understanding of drug-target interactions.

In the context of predicting drug toxicity, several models have been developed in recent years, driven by the importance of drug–protein interactions in understanding drug toxicity mechanisms and the increasing focus on target-based drug development. TargeTox, which was introduced in 2018, employs an integrative ML approach that uses information about all proteins that can bind a drug, including both intended pharmacological targets and off-targets, to improve the prediction of toxicity-related drug safety. It can be used to differentiate potentially idiosyncratic toxic drugs from safe drugs.^122^ Chua et al. contributed to this field by developing an approach to predict synergistic target combinations from curated signaling networks, named MASCOT. This method represents an intersection of ML, systems biology, and pharmacology.^123^ Moreover, a recent study investigated the comprehensive targets of bisphenol A and its associated pathway, potentially contributing to the observed adverse outcomes and elucidating the toxic pathogenic effects.^124^ These models play a pivotal role in bridging the gap between toxicity and DTBA, aiming to enhance the prediction of toxicity. A recent review highlighted the significance of computational modeling in nanotoxicology, including systems biology and bioinformatics. This emphasizes the use of AI to analyze toxicology data sets and develop physiologically based pharmacokinetic and nanoquantitative structure–activity relationship models. This review also discusses toxicogenomics, which investigates the genetic basis of toxic responses in living organisms.^125^ Another review highlighted the need for a multidisciplinary approach to revolutionize toxicology. This involves refining our understanding of toxicology, predicting potential risks, and developing treatment modalities.^12^ However, the development of such models, including the effective representation of a multifaceted issue in vitro, in vivo, and clinical platforms, is challenging. Considering that the in vivo relevance of drug target binding is crucial in predicting drug toxicity, the pharmaceutical industry is increasingly employing computational and integrative approaches to address this limitation. Therefore, further research and development of better methods to assess drug toxicity are required. Integrating omics data,^126−128^ explainable pharmacological data, and features,^129^ and refining in silico modeling through AI, collaboration, and data sharing will be pivotal in the future. Addressing these challenges will advance the field, leading to more reliable predictive models, and consequently, safer drug development practices in the pharmaceutical domain. Table 2 presents a comprehensive comparison of the methods described above.

**Table 2:** Summary of Performance of State-of-the-Art Models for DTBA Prediction^a^

authors	name	method type	data set	data set size/interactions	target variable	R^2^	r_m_^2^	RMSE	SCC	PCC	CI
(116)	KronRLS	ML	Davis	30 056 (68 ligands and 442 targets)	*K*_d_	0.580	0.407^d^	0.573			0.883
						0.048^2, 4^		0.840^2,4^			0.748^e^
						0.439^3,4^		0.660^3,4^			0.861^f^
			KIBA	118 254 (2116 drugs and 229 proteins)	KIBA score	0.413	0.342^1^	0.657			0.792^1^
						0.327^2, 4^		0.702^2,4^			
						0.363^3,4^		0.681^3,4^			
			Metz	35 259 (1.421 compounds and 156 kinases)	*K*_i_	0.335		0.781			0.793
						0.328^2, 4^		0.784^2,4^			0.736^2^
						0.113^3,4^		0.899^3,4^			0.666^3^
(90)	SimBoost	ML	Davis	30 056 (68 ligands and 442 targets)	*K*_d_	0.703^g^	0.644^1^	0.247			0.884
			KIBA	118 254 (2116 drugs and 229 proteins)	KIBA score	0.701^4^	0.629^1^	0.204			0.847
			Metz	35 259 (1421 compounds and 156 kinases)	*K*_i_	0.632^4^		0.116			0.851
(96)	PADME	Graph-based	Davis	30 056 (68 ligands and 442 targets)	*K*_d_	0.765		0.429			0.903
						0.144^2^		0.805^2^			0.712^2^
						0.591^3^		0.564^3^			0.854^3^
			KIBA	118 254 (2116 drugs and 229 proteins)	KIBA score	0.745		0.433			0.858
						0.509^2^		0.601^2^			0.774^2^
						0.471^3^		0.623^3^			0.768^3^
			Metz	35 259 (1,421 compounds and 156 kinases)	*K*_i_	0.665		0.556			0.806
						0.448^2^		0.712^2^			0.743^2^
						0.318^3^		0.790^3^			0.696^3^
(77)	DeepDTA	DL	Davis	30 056 (68 ligands and 442 targets)	*K*_d_		0.630	0.511			0.878
			KIBA	118 254 (2116 drugs and 229 proteins)	KIBA score		0.673	0.440			0.863
			BindingDB	263 534 training samples and 113 142 test samples	*K*_i_			0.686^h^		0.886^5^	
(117)	WideDTA	DL	Davis	30 056 (68 ligands and 442 targets)	*K*_d_			0.512		0.820	0.886
			KIBA	118 254 (2116 drugs and 229 proteins)	KIBA score			0.423		0.856	0.875
(119)	IVPGAN	DL	Davis	30 056 (68 ligands and 442 targets)	*K*_d_	0.945		0.201			0.973
						0.863^2^		0.289^2^			0.949^2^
						0.906^3^		0.220^3^			0.963^3^
			KIBA	118 254 (2116 drugs and 229 proteins)	KIBA score	0.766		0.400			0.843
						0.647^2^		0.470^2^			0.807^2^
						0.706^3^		0.449^3^			0.823^3^
			Metz	35 259 (1421 compounds and 156 kinases)	*K*_i_	0.628		0.553			0.791
						0.617^2^		0.548^2^			0.789^2^
						0.593^3^		0.574^3^			0.778^3^
(92)	GANsDTA	DL	Davis	30 056 (68 ligands and 442 targets)	*K*_d_		0.653	0.525			0.881
			KIBA	118 254 (2116 drugs and 229 proteins)	KIBA score		0.675	0.473			0.866
(118)	MT-DTI	DL	Davis	30,056 (68 ligands and 442 targets)	*K*_d_		0.665	0.495			0.887
			KIBA	118 254 (2,116 drugs and 229 proteins)	KIBA score		0.738	0.390			0.882
(93)	DGraphDTA	Graph-based	Davis	30 056 (68 ligands and 442 targets)	*K*_d_		0.700	0.450		0.867	0.904
			KIBA	118 254 (2116 drugs and 229 proteins)	KIBA score		0.786	0.355		0.903	0.904
(120)	MONN	DL	BindingDB	263 534 training samples and 113 142 test samples	*K*_i_			0.658		0.895	
(94)	GraphDTA	Graph-based	Davis	30 056 (68 ligands and 442 targets)	*K*_d_			0.478			0.893
			KIBA	118 254 (2116 drugs and 229 proteins)	KIBA score			0.373			0.891
(81)	Affinity2Vec	Graph-based	Davis	30 056 (68 ligands and 442 targets)	*K*_d_		0.693	0.490			0.887
			KIBA	118 254 (2116 drugs and 229 proteins)	KIBA score		0.765	0.352			0.910
(115)	DeepMHADTA	DL	Davis	30 056 (68 ligands and 442 targets)	*K*_d_		0.701	0.494			0.895
			KIBA	118 254 (2116 drugs and 229 proteins)	KIBA score		0.719	0.431			0.876
(121)	WGNN-DTA	Graph-based	Davis	30 056 (68 ligands and 442 targets)	*K*_d_			0.456		0.863	0.898
			KIBA	118 254 (2116 drugs and 229 proteins)	KIBA score			0.360		0.900	0.884

a Abbreviations: R^2^: Coefficient of Determination, r_m_^2^: Modified r^2^ ;^b^ RMSE: Root Mean Square Error, SCC: Spearman Correlation Coefficient, PCC: Pearson Correlation Coefficient, CI: Concordance Index^c^, ML: Machine Learning, *K*_d_: Dissociation Constant, KIBA: Kinase Inhibitor BioActivity, *K*_i_: Inhibition Constant, DL: Deep Learning.

b r_m_^2^ considers the actual difference between the observed and predicted response data without considering the training set mean.^130^

c The CI is a generalization of the AUC and measures the ability of a model to correctly rank the survival times of individuals. It ranges from 0 to 1, where a higher value indicates better predictive performance.^131^

d Results retrieved from DeepDTA.^77^

e These were obtained under the S2 or cold drug condition; that is, only the drug was not encountered in the training set.

f These were obtained under the S3 or cold target condition; that is, only the target was not encountered in the training set.

g Results retrieved from PADME.^96^

h Result obtained from Li et al.^120^

## 5 In Vivo Pharmacokinetics: Enhancing Prediction and Safety

Recognizing the limitations inherent in toxicity prediction models, it is important to note that the aforementioned toxicity end points, although widely used and informative, often merely serve as proxy indicators that may not fully capture the multifaceted nature of biological toxicity in humans. A relevant area of focus in pharmacology relies on in vivo PK predictions, which reflect a deeper understanding of how drugs interact with living organisms, including at all stages of ADME. The adoption of artificial intelligence (AI) in this sphere, which leverages PK data to forecast toxicity, marks a significant evolution in our approach to drug safety.^132^ Innovations such as in vitro to in vivo extrapolation (IVIVE) techniques and allometric scaling have been instrumental in translating PK data into human scenarios, enhancing our ability to mitigate drug administration risks.^133^ IVIVE utilizes mathematical modeling and can be used to predict in vivo phenomena, including their concentrations and effects. For instance, a study on the anticancer drug oxaliplatin aimed to integrate ex vivo PK data from mice, rats, and humans to model the behavior of the drug within whole blood. Researchers then extended these findings to predict the whole-body pharmacokinetics in humans.^134^ However, performing such intricate and expensive experiments on a vast array of compounds is impractical.^135^ Physiologically Based Pharmacokinetic (PBPK) models^136,137^ supporting IVIVE data have become a cornerstone in translating laboratory findings to clinical applications, as demonstrated by research combining Siremadlin and Trametinib against melanoma. Utilizing PBPK modeling and virtual trials, this approach integrates laboratory, animal, and clinical insights, highlighting the potential of in vitro to in vivo extrapolation for refining cancer treatments.^138^ Furthermore, such methodologies contribute to the implementation of the 3Rs (replacement, reduction, and refinement).

A significant obstacle in constructing these models is the scarcity of the necessary drug/chemical-specific parameters, for which measurements often remain unavailable,^139^ a gap that AI has begun to fill.^135,139,140^ AI models can be developed by utilizing the available PK data, and their incorporation into PBPK models facilitates the prediction of PK parameters.^139^ The inclusion of ML in PBPK models has improved their performance, offering a sophisticated approach for understanding drug kinetics and safety.^141^ This integration represents a promising framework to overcome the limitations of extrapolating data, particularly for predicting complex biological interactions and individual variability in drug responses. Several reviews on this subject have been published in recent years, containing detailed information on PK parameters, databases, and integration of ML and PBPK, serving as a testament to the growing interest in and potential of this approach.^132,135,139,140^ The drug parameters discussed for drug safety include the maximum plasma concentration (*C*_max_), which should be substantially lower than the maximum tolerated concentration (MTC), to minimize the risk of adverse effects. However, for efficacy, the drug concentration must remain at or above the minimum effective concentration (MEC) for a sufficient duration. Furthermore, the desired drug should exhibit high bioavailability (*F*) in oral or subcutaneous deliveries to ensure optimal in vivo exposure and preferably low clearance (CL), extending the duration of the therapeutic effect of the drug.^132^ Attention should be paid to the integration of AI into PK modeling to enhance its predictions, as AI can deal with large data sets and potentially identify relevant patterns.

PBPK modeling and therapeutic drug monitoring (TDM) represent two pivotal strategies for optimizing drug dosing regimens and ensuring both the safety and efficacy of therapeutics. TDM is a traditional yet equally critical medical practice that involves the measurement of specific drug concentrations in a patient’s biological fluid at scheduled intervals.^142^ TDM is particularly important for drugs with a limited therapeutic range. Furthermore, PBPK and its ability to simulate drug behavior in the body are particularly beneficial for drugs with a narrow therapeutic index (TI).^143^ In vivo PK studies are crucial for estimating TI, a measure comparing the drug concentration in the blood that leads to toxicity with the concentration that produces therapeutic benefits. The index is defined as the ratio between LD50 and the effective dose (ED50), with LD50 indicating the level of toxicity and ED50 indicating efficacy. A higher TI indicates increased safety, whereas a lower TI suggests a narrow margin. Moreover, in clinical settings, drugs with a narrow TI can shift from therapeutic to toxic even with small variations in dose or blood concentration, necessitating precise dosing and vigilant monitoring.^144^ Researchers and healthcare providers need to balance efficacy and safety to ensure that the benefits of a drug are greater than the potential risks. The LD50/ED50 ratio aids in the evaluation but has limitations. For instance, LD50 values are derived from animal models and may not translate directly into humans. Another challenge is that efficacy and safety depend on individual variations. Consequently, the TI may not accurately reflect the true risk profile of a drug. Additionally, genetic differences can affect drug metabolism, efficacy, and probability of adverse reactions.^145^

In light of this, the exploration and integration of TI with PBPK modeling and AI represents a promising approach for optimizing drug dosing regimens for enhanced safety and efficacy. By embracing a multidisciplinary perspective, this approach aims to overcome the limitations of conventional toxicity prediction, thereby offering a more personalized medicine trajectory. The strategic use of extensive data sets, including genetic information, heralds a future in which drug dosing is customized to the unique profiles of individual patients, markedly diminishing the hazards linked to limited TIs and diverse patient responses. The enhancement of PBPK models through AI integration is poised to address challenges related to the accurate interpretation of pharmacokinetic data, facilitating the development of safer and more potent therapies. Moreover, it is imperative to focus on the clarity and reliability of AI-generated pharmacokinetic predictions to ensure that they undergo continuous improvement and validation. Utilizing data from in vivo animal studies and applying transfer learning techniques to apply this knowledge to constrained human data sets could prove to be a fruitful approach. Dedicated research is essential to fully leverage these combined methodologies, concentrating on identifying specific drug-related factors and fine-tuning therapeutic strategies.

## 6 Conclusion and Future Perspectives

The ability to accurately predict drug toxicity during the preclinical stage of development is paramount for several reasons. First, it serves as a critical filter to identify compounds that may pose health risks, thereby safeguarding patient safety and avoiding the potential for adverse effects that could emerge in the later stages of clinical trials or postmarket. The early detection of toxicity can substantially reduce the financial and ethical costs associated with the development of drugs that may ultimately prove unsafe for human use. Furthermore, there is an increasing need to enhance the adoption of in silico models for toxicological predictions. In silico methods have several advantages. They can process vast chemical libraries rapidly and at a fraction of the cost compared with traditional experimental approaches. Additionally, in silico models have the potential to uncover toxicological end points that are difficult to measure in vivo or in vitro, providing a more comprehensive safety profile for the candidate drugs. Finally, the application of in silico predictions aligns with the principles embodied in the 3R guidelines: replacement, reduction, and refinement. Using computational models, researchers can replace the need for live animal testing, thus adhering to the ethical imperative of reducing the use of animals in research. When in vivo testing is unavoidable, in silico models can refine the process by identifying the most promising compounds, thereby minimizing the number of animals required for testing. This compliance not only reflects an ethical commitment to animal welfare, but also supports the scientific community’s responsibility to conduct research in a humane, responsible manner. Hence, to address these challenges effectively, pharmaceutical companies are increasingly turning to computational methods, particularly AI-based approaches, to streamline the toxicity prediction process and enhance productivity. These AI algorithms have proven valuable in the early detection of potentially harmful substances during drug discovery. Several ML/DL-based methods, including LD50, DILI, hERG inhibition, carcinogenesis, and Ames mutagenesis, have been developed to more efficiently predict various toxicity end points. Advances in computational technology have significantly improved the drug development process, benefiting both the pharmaceutical industry and patient health. DTBA plays a pivotal role in toxicity assessments and is a vital factor for evaluating drug safety. It is crucial not only to determine a drug’s effectiveness, but also to predict potential adverse effects and toxicity, underlining its importance in comprehensive drug safety assessments.

We believe that, throughout the drug discovery journey, the detailed insights provided in Tables 1 and 2 offer a comprehensive and targeted perspective at different stages of the process, adding value to the current scientific landscape. Table 1, comprising diverse toxicity prediction models and performance metrics, plays a pivotal role in identifying potential adverse effects. In parallel, Table 2 presents a diverse array of models and performance metrics for DTBA prediction. This study explores DTIs, acting as a guide for researchers and shedding light on intricate details that direct the choice and optimization of promising contenders. These tables not only enhance researchers’ understanding of essential drug characteristics, but also provide a considerable edge in drug discovery. By incorporating them, a comprehensive strategy is established, streamlining the drug development process, and promoting a reliable and secure approach.

The field of drug toxicity prediction is poised for substantial advancements, driven by technological progress and increased data availability. The development of new models capable of delivering more informative toxicity metrics is required. These models must effectively process experimental data and demonstrate their relevance in real-world scenarios for accurate toxicity predictions. They are designed to be efficient and require minimal computational resources to manage the extensive chemical libraries. However, several challenges must be addressed, including enhancing model interpretability, addressing gaps in toxicity knowledge, and navigating the complexity of biological systems. Additionally, improving the quality of the models and data is crucial. A key area of potential breakthroughs lies in understanding the relationship between toxicity and DTBA. Future research should focus on developing models that are not only interpretable and robust, but also capable of integrating diverse data sources. This comprehensive approach is essential for advancing drug toxicity predictions and ensuring its relevance to in vivo studies and clinical applications.
